# Influence of sample-specific properties on light scattering by human milk

**DOI:** 10.1117/1.BIOS.3.1.012105

**Published:** 2026-03-09

**Authors:** Wietske Verveld, Johanna Rebecca de Wolf, Nienke Bosschaart

**Affiliations:** a University of Twente, Biomedical Photonic Imaging Group, Faculty of Science and Technology, Enschede, The Netherlands

**Keywords:** scattering, human milk, optical properties, Mie theory, particle sizing, refractive index.

## Abstract

**Significance:**

Despite the numerous benefits of breastfeeding, many mothers quit breastfeeding early due to understudied lactation problems. Optical and photonic techniques provide promising, objective, and non-invasive methods for human milk analysis but require accurate characterization and modeling of light scattering in human milk.

**Aim:**

We aimed to describe the influences of sample-specific properties on the forward scattering coefficient of human milk samples.

**Approach:**

We composed an experimental dataset of 50 human milk samples, containing the scattering coefficient, fat concentration, serum refractive index, milk fat globule (MFG) refractive index, MFG size distribution, and refractive index and size distribution of non-fat particles per sample. We reconstructed scattering coefficients using Mie theory with sample-specific properties and compared them to experimental values.

**Results:**

This study confirmed a moderate correlation between experimental and reconstructed scattering coefficients when only the sample-specific fat concentration was considered (Spearman ρ=0.62). Including the sample-specific MFG size distribution significantly improved the correlation (ρ=0.82), while the sample-specific scattering contribution of non-fat particles and refractive index of serum and MFGs had a negligible influence.

**Conclusions:**

The sample-specific fat concentration and MFG size distribution are the most important parameters to consider when developing light scattering models of human milk.

Statement of Discovery This work compared the scattering coefficient of human milk samples from collimated transmission with reconstructions based on Mie theory and a broad dataset of sample-specific properties. We discovered that the sample-specific fat concentration and milk fat globule size distribution are the most important parameters to consider when developing light scattering models of human milk, which could be applied for non-invasive studies on human lactation.

## 1 Introduction

Breastfeeding offers numerous health benefits to both infants and mothers,^1^^,^^2^ along with economic and environmental benefits for families and society.^3^ Therefore, the World Health Organization recommends exclusive breastfeeding for the first 6 months of infant life.^4^ In recent years, this goal has been met only by ∼44% of mothers worldwide.^5^ Regardless of the age of weaning, the main reason to stop breastfeeding earlier than desired is perceived lactation insufficiency for 40% to 60% of mothers.^6^^,^^7^ Interestingly, the estimated incidence of actual lactation insufficiency is estimated to be much lower, at 10% to 15%.^8^ To better understand the causes of lactation problems, objective and non-invasive methods to measure human milk intake and composition are necessary.

A milk property of specific interest for determining the total nutritional value of human milk is the fat concentration, as fat is the main energy source in human milk.^9^ However, the fat concentration in human milk is highly sample-dependent, with reported values ranging from ∼0.5 to 10  g/dL.^10^^–^^14^ Fat concentrations commonly increase more than twofold within one breastfeed, from the foremilk (initial milk that is secreted during a breastfeed) to the hindmilk (last fraction of secreted milk). Nevertheless, the hindmilk of some mothers can contain less fat than the foremilk of other mothers. Therefore, accurate fat quantification is necessary to estimate the total energy intake of an individual baby. This is of particular interest for the purpose of target milk fortification in premature infants.^15^ The quantification of fat concentrations can be of additional interest during secretory activation (3 to 8 days postpartum), as it may serve as a potential marker for successful lactation.^16^

Lipids in human milk are contained in milk fat globules (MFGs). An MFG has a lipid core with triacylglycerol molecules, surrounded by a triple-layered phospholipid membrane. Human MFGs can be considered spherical, with diameters ranging from 0.5 to 15  μm.^17^^,^^18^ These micron-sized fat globules are the main light scattering particles in human milk in the visible and near-infrared spectral range, because of the high refractive index contrast between the lipids (∼1.506 at 405 nm^19^) and the surrounding milk serum (∼1.3470 at 589 nm^20^). This suggests that optical or photonic light scattering techniques could offer promising methods for the characterization and quantification of milk fat. A light scattering-based method for fat quantification can potentially overcome the drawbacks of current methods, such as low accuracy in creamatocrit measurements,^21^ the labor-intensiveness of biochemical methods,^22^ and the homogenization requirement for infrared absorption spectroscopy.^23^ In addition, optical characterization could ideally suffice with small sample volumes, which would benefit research into situations with limited milk availability, such as lactation insufficiency or preterm milk at the neonatal intensive care unit.^24^

For the development of optics-based measurement systems for human milk, accurate characterization and modeling of light scattering by human milk are essential. In our previous work, the bulk optical parameters of human milk were characterized using diffuse reflectance spectroscopy and spectroscopic optical coherence tomography.^13^ Although the scattering coefficient $\mu_{s}$ and reduced scattering coefficient $\mu_{s}^{\prime }$ correlated significantly with milk fat concentration, these scattering properties alone turned out to be insufficient for accurate quantification of milk fat concentration. This only moderate predictive value of $\mu_{s}$ and $\mu_{s}^{\prime }$ was hypothesized to be caused by biological variations in milk properties.

Several sample-specific milk properties are expected to influence the scattering behavior in human milk. Theoretically, the scattering cross-section for individual, homogeneous spherical particles with a known size and any refractive index contrast to the surrounding medium at a given wavelength is described by Mie theory.^25^ If the particle concentration is known, the forward scattering coefficient of a sample can be derived. In the case of human milk samples, we expect large variations in the scattering coefficient due to the wide biological range of milk fat concentrations. In addition, the size distribution of MFGs is expected to have a significant influence on light scattering in human milk. Multiple studies have shown that the mean size of MFGs varies significantly with lactation stage, moment within breastfeeding, and maternal physiology.^13^^,^^17^^,^^26^^–^^28^ Further, we have recently shown that the refractive indices of human milk serum and MFGs are sample-dependent.^19^^,^^20^ Finally, although MFGs are the predominant scattering particles in human milk, other scattering particles such as casein micelles, extracellular vesicles, and cells occur in variable and sample-specific concentrations.^20^^,^^29^ These non-fat particles could also cause a sample-specific contribution to the scattering behavior of human milk. Currently, it is unclear what the specific influence of all aforementioned sample-specific properties is on the light scattering behavior by human milk.

In this study, we aimed to quantify the influence of these sample-specific milk properties on the scattering coefficient of human milk. To this end, we compared experimental scattering coefficients with reconstructed scattering coefficients from Mie theory, using either mean or sample-specific input. Sample-specific input involved measurements of the fat concentration, serum refractive index, MFG refractive index, MFG size distribution, and non-fat particle refractive index and size distribution per milk sample. This approach will provide in-depth insight into the milk properties that need to be taken into account when developing light scattering-based methods for human milk fat quantification.

## 2 Materials and Methods

**Fig. 1:**
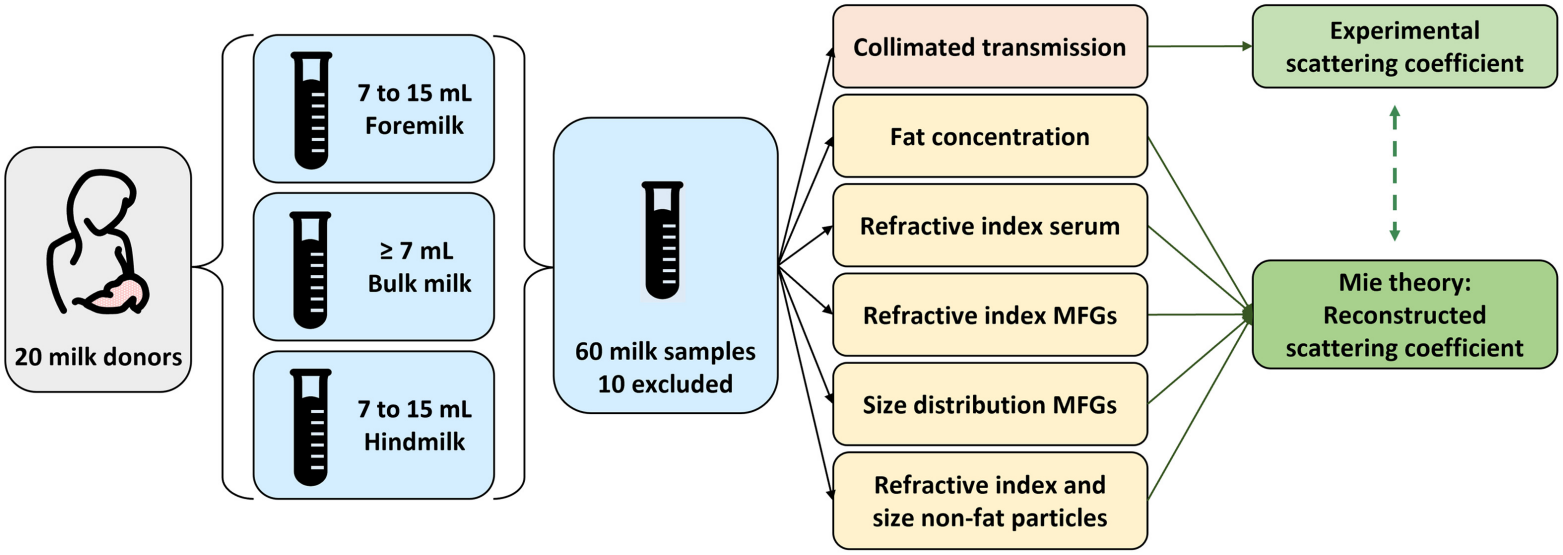
Schematic overview of the study methods. Twenty healthy lactating women donated milk from one pumping session in three parts: foremilk, bulk milk, and hindmilk. Out of the 60 milk samples in total, 10 were excluded (see Sec. 2.1). Milk samples were analyzed with various setups to obtain sample-specific values of the fat concentration, refractive index of the serum, refractive index of the MFGs, size distribution of MFGs, and refractive index and size distribution of non-fat particles. The experimental scattering coefficients from collimated transmission measurements were compared with reconstructed scattering coefficients based on the sample-specific properties and Mie theory.

An overview of the methods that were followed in this study is given in Fig. 1. We first explain the human milk sample collection and characterization of the sample-specific milk properties in Secs. 2.1 and 2.2, respectively. Next, two methods to obtain scattering coefficients are described: collimated transmission for experimental results (Sec. 2.3.1), and reconstructions with Mie Theory to study the effects of individual sample-specific properties (Sec. 2.3.2). Finally, the statistical analysis is addressed (Sec. 2.4).

### 2.1 Sample Collection

Twenty healthy breastfeeding women aged between 27 and 39 years with a lactation period between 0.8 and 9 months postpartum donated human milk samples for this study and our related earlier work.^14^^,^^18^^–^^20^^,^^28^ The milk was collected from one breast during a single pumping session in three fractions: foremilk (the first 7 to 15 mL), bulk milk (middle fraction), and hindmilk (the last 7 to 15 mL). Milk samples were divided into volumes of 150  μL to 3 mL for the different analysis techniques before freezing at –20°C. Milk was frozen within 15 h after donation and analyzed between 2 and 17 months after donation. Freezing and storage were expected to have a minimal effect on the total fat content^30^ but could lead to aggregation of MFGs.^31^^,^^32^ Therefore, milk analysis with the various methods was conducted with as much temporal proximity as was practically achievable. Out of the 60 milk samples in total, 10 were excluded due to insufficient sample volume for analysis or poor milk quality with visible flocculation after a freeze-thaw cycle.

Ethical approval was obtained from the Natural Sciences and Engineering Sciences Ethics Committee of the University of Twente (reference no. 2021.118). All participants gave written informed consent before milk donation.

### 2.2 Sample-Specific Properties

For each included milk sample, the following sample-specific properties were determined: fat concentration, serum refractive index, MFG refractive index, MFG size distribution, and non-fat particle refractive index and size distribution. These properties together provide a complete dataset with all the required medium and scattering particle information for reconstruction of the scattering coefficients using Mie theory, as described hereafter (Sec. 2.3.2).

#### 2.2.1 Fat concentration

The macronutrient concentrations, including the fat concentration, of all milk samples were measured as previously described^14^ with a human milk analyzer (HMA, Miris AB, Uppsala, Sweden). The HMA is based on mid-infrared spectroscopy, with a reported accuracy of ±10% for fat concentration measurements.^33^ For these measurements, milk samples of 3 mL were thawed for at least 30 min at room temperature and subsequently heated for 30 min at 40°C in a heater bath (Miris AB, Uppsala, Sweden) according to the HMA measurement protocol.^33^ The HMA measurement protocol was also followed for subsequent milk sonification, measurements, and cleaning. Hind milk samples were diluted 1:1 with Milli-Q^®^ ultrapure water before homogenization, because the expected fat concentration was above the recommended measurement range of the HMA (0.6 to 4  g/dL).^33^ The resulting fat concentrations were corrected for this dilution step.

#### 2.2.2 Refractive index milk serum

Milk serum, sometimes also referred to as whey, is defined as the liquid medium in which MFGs and other milk particles are suspended.^20^ The refractive index of the milk serum, $n_{\text{serum}}$, was measured at 589.3 nm and room temperature with a refractometer (Mettler Toledo Refracto 30GS, Columbus, Ohio, United States) after skimming each donated milk sample, as described in detail in our previous work.^20^ The refractive index was converted from 589.3 to 660 nm with an absolute difference of −0.0019. This is the same absolute difference as between the refractive indices of water for these wavelengths at 21.5°C.^34^ For context, biofluids such as cytoplasm, blood serum, and cell media have been shown to have similar chromatic dispersion.^35^^–^^37^

#### 2.2.3 Refractive index milk fat globules

The refractive index distribution of the MFGs with a diameter between 200 and 650  nm was measured using flow cytometry (FCM) at 405 nm, as described in our previous work.^19^ The sample-specific MFG refractive index distribution in the current study is reduced to one value per sample, $n_{\mathrm{MFG}}$, defined as the mean of the MFG refractive index distribution. As the dispersion of human milk fat is unknown, the refractive index was converted from 405 to 660 nm with an absolute difference of −0.016. This is the same absolute difference as between the refractive indices of bovine milk fat for these wavelengths.^38^

#### 2.2.4 Size distribution milk fat globules

The size distribution of the MFGs in each milk sample was measured using three-dimensional (3D) confocal laser scanning microscopy.^18^ In short, 150  μL milk was thawed at room temperature, stained with Nile Red for 15 min, and fixed with agarose gel before imaging. 3D image stacks were processed automatically to obtain a list of all diameters in the image stack. This method was shown to be accurate for human MFGs between 500 nm and 10  μm in diameter. In addition to the protocol in Ref. 18, we manually segmented the few larger fat globules that partly crossed the image stack borders to increase the upper detection limit, as described in Ref. 28. The list of all diameters found per sample was converted to a number distribution with 1000 linearly scaled diameter bins between 0 and 50  μm. A mean size distribution was obtained for all 50 included samples after normalizing the size distributions. The sample-specific fat concentration and a fat density of 0.915  kg/L^39^ were used to calculate the number of MFGs per bin per liter of human milk, $N_{\mathrm{MFG}}(d)$, where d is the MFG diameter. For visualization, the size distributions were converted to a normalized number distribution with 100 logarithmically scaled diameter bins between 0.1 and 1000  μm.

#### 2.2.5 Refractive index and size distribution non-fat particles

The refractive index and the size distribution of non-fat particles were also measured using FCM, as described in our previous work.^19^ In the current study, we included all non-fat particles with FCM signals above the noise limit, a diameter between 200 and 650  nm, and a refractive index below 1.55. The refractive index was converted from 405 to 660 nm with an absolute difference of −0.0116. This is the same absolute difference as between the refractive indices of water for these wavelengths at 21.5°C.^34^

Per milk sample, a two-dimensional histogram was formed with 200 and 100 linearly spaced bins for the diameter and refractive index, respectively. The incidence of non-fat particles per bin with particle diameter d and refractive index $n_{p}$ was converted to a number per liter distribution $N_{\text{non-fat}}(d,n_{p})$, based on the dilutions used in the FCM measurements. The arithmetic mean for all 50 included samples was calculated per histogram bin.

### 2.3 Light Scattering Analysis

Next, various reconstructed scattering coefficients were computed using either the sample-specific or mean properties. The reconstructed scattering coefficients were compared with the experimental scattering coefficient of each milk sample.

#### 2.3.1 Experimental scattering coefficient

**Fig. 2:**
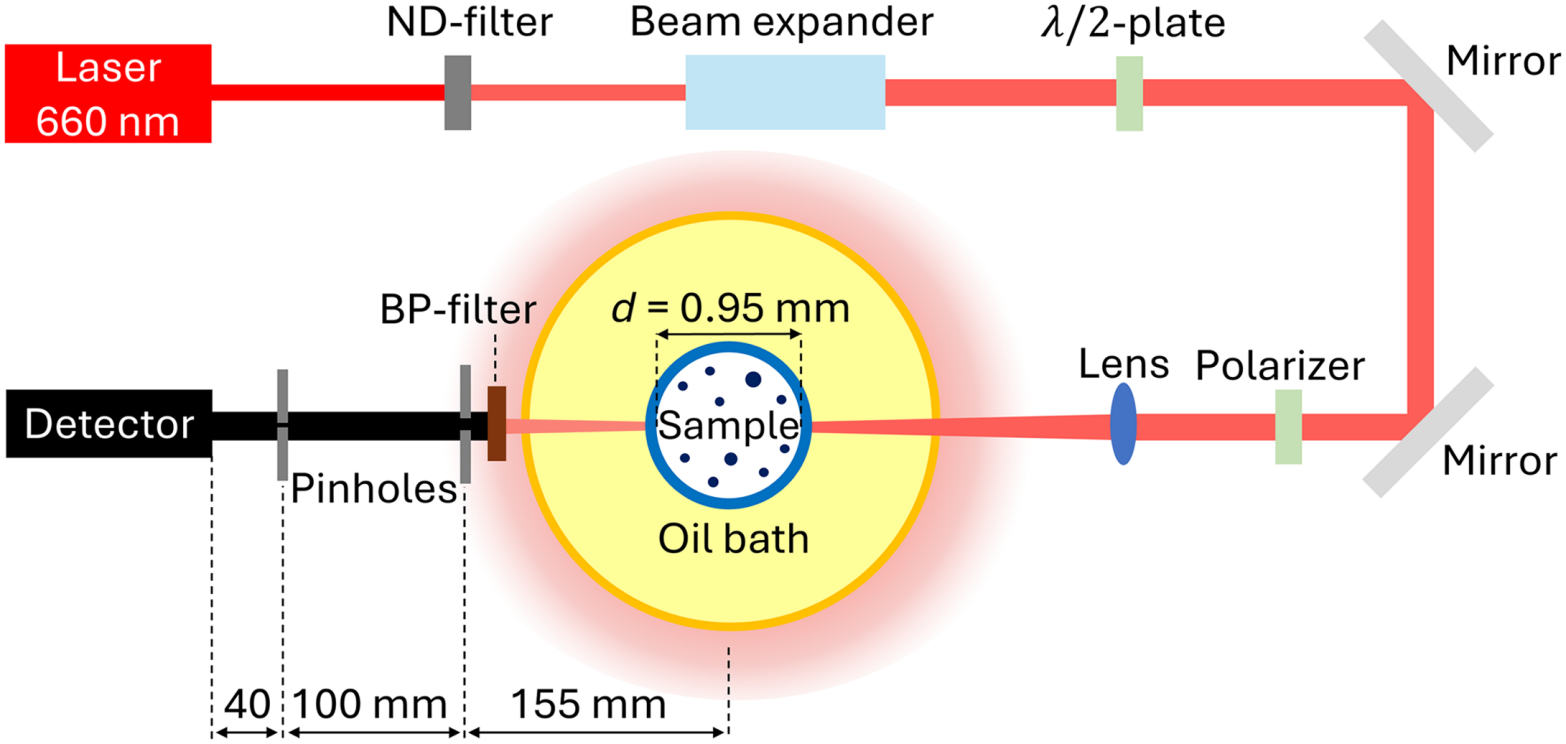
Simplified schematic of the goniometric light scattering setup in forward collimated transmission geometry that was used to obtain experimental scattering coefficients. The sample (human milk or water) was placed in a 0.95-mm diameter capillary tube, and two pinholes were positioned before the detector to limit the angle of incident light.

Experimental scattering coefficients were obtained from collimated transmission measurements. For this study, the transmitted light intensity in water and human milk was measured with a goniometric light scattering setup in the forward direction only (Fig. 2). The setup consisted of a continuous wave diode-pumped laser with a center wavelength of 660 nm (Cobolt Flamenco 50 mW, Cobolt AB, Solna, Sweden), neutral density (ND) filters with optical densities between 0.2 and 9.6, a beam expander (Spatial filter and Collimator 25 mm, Melles Griot, United States), a λ/2 wave-plate (RSP1X15/M, Thorlabs, Newton, New Jersey, United States), mirrors on kinematic mirror mounts for alignment, a polarizer (LPVIS100MP, Thorlabs, Newton, New Jersey, United States) to select the horizontal polarization parallel to the optical table, and a lens (AC254-400-A-ML, Thorlabs, Newton, New Jersey, United States). The incoming laser bundle was focused at the center of the milk sample. The small convergence of the beam diameter with 3.30  mm/m in air corresponded to a Rayleigh length of ∼2  cm, which is sufficiently long compared with the sample diameter to classify the system as a collimated transmission setup. A single-photon counter module detector (SPCM-AQR-13, PerkinElmer, Waltham, Massachusetts, United States) was placed behind a 661/11 bandpass (BP) filter and two pinholes of 1 mm and 400  μm diameter at distances of 140 and 40 mm from the detector, respectively (Fig. 2). These pinholes limited the angle of incident light on the detector to 0.05 deg and thereby reduced the detection of multiple scattered photons. The samples were placed in a round capillary tube (HIRS9201360, Hirschmann Laborgerate, Eberstadt, Germany) with a 0.95-mm inner diameter. This tube was placed in a 65-mm borosilicate glass bath (HL692-455-23, Hellma Analytics, Müllheim, Germany) filled with transparent baby oil (Trekpleister baby huidolie, Trekpleister, Renswoude, The Netherlands) to minimize reflections.

For these measurements, milk samples of 150  μL were thawed for at least 1 h at room temperature. The samples were gently mixed before being transferred into a capillary tube. The bottom of the tube was then sealed with hematocrit sealing wax (Glaswarenfabrik Karl Hecht, Sondheim vor der Rhön, Germany), and the sample was measured directly thereafter to minimize the effects of creaming and sedimentation. For each capillary tube, the mean photon intensity I over 3 s in the forward direction was used as the transmission intensity value in this study. Each milk sample was measured three times in new capillary tubes. Transmission reference values were measured in triplicate with Milli-Q^®^ ultrapure water.

The output count rate per second (cps) from the single photon counter module was corrected for the module dark count rate ${\mathrm{cps}}_{\text{dark}}$, the module photon detection efficiency η, and a correction factor $c_{f}$ based on the module dark time $t_{d}$ and output count rate, using Eqs. (1) and (2).^40^

$$I=\frac{\mathrm{cps}\cdot c_{f}-{\mathrm{cps}}_{\text{dark}}}{\eta },$$ (1)

$$c_{f}=\frac{1}{1-t_{d}\cdot \mathrm{cps}}.$$ (2)

${\mathrm{cps}}_{\text{dark}}$ was determined experimentally. η and $t_{d}$ were obtained from the detector manual.^40^ The scattering coefficient $\mu_{s}$ was used to compare measurements and reconstructions with various sample-specific properties. The experimental scattering coefficients were obtained with Eq. (3), which was derived from the Lambert–Beer law in a turbid medium:^41^

$$\mu_{s,\text{Experiment}}=-\text{ln}\left(\frac{I_{\text{Sample}}}{I_{\text{Water}}}\right)/d_{\text{capillary}},$$ (3)

where $d_{\text{capillary}}$ is the capillary inner diameter, $I_{\text{Sample}}$ is the mean transmitted intensity through the sample of interest, and $I_{\text{Water}}$ is the reference transmitted intensity through ultrapure water. Error margins (95% confidence interval) for $\mu_{s,\text{Experiment}}$ are propagated from the standard deviation of three measurements.

The Lambert–Beer law, which underlies Eq. (3), assumes that only ballistic light is detected. This assumption fails for highly scattering samples where multiple scattering becomes dominant (>10 scattering events per path length). In our setup with a sample diameter of 0.95 mm, experimental scattering coefficients above $∼9.5\;{\mathrm{mm}}^{-1}$ could not be measured reliably due to multiple scattering. For milk samples where the result exceeded a limit of $8\;{\mathrm{mm}}^{-1}$, we therefore diluted the milk with ultrapure water by a factor of 5 and 10 and repeated the measurements in triplicate for both dilutions. Based on preliminary experiments, moderate dilution with ultrapure water changed the scattering coefficient of human milk linearly as expected. The experimental scattering coefficients from the additional measurements with diluted milk were extrapolated to a new experimental scattering coefficient as if it were undiluted milk, using a linear fit through the origin with the function fit in Matlab (R2024a, The MathWorks Inc., Natick, Massachusetts, United States). The error margins for the extrapolated scattering coefficients were propagated from the 95% confidence interval of the linear fit of six diluted measurements.

#### 2.3.2 Reconstructed scattering coefficient

Various reconstructed scattering coefficients were computed to study the influence of individual and combined sample-specific properties. The reconstructed scattering coefficient of a milk sample was calculated as the summation of the scattering contributions from all individual particles, using

$$\mu_{s,\text{Reconstructed}}=\underset{d,n_{p}}{\sum }[N(d,n_{p})\sigma_{s}(d,n_{p},n_{m},\lambda )],$$ (4)

$$N(d,n_{p})=N_{\mathrm{MFG}}(d,n_{p})+N_{\text{non-fat}}(d,n_{p}),$$ (5)

where $N(d,n_{p})$ is the total number distribution per milk volume of both MFGs and non-fat particles, combined from Secs. 2.2.4 and 2.2.5, respectively. For the MFGs, one refractive index $n_{p}=n_{\mathrm{MFG}}$ was used per milk sample. In contrast, a particle-specific refractive index was used for all non-fat particles, due to the larger variation between different types of non-fat particles. The scattering cross-section $\sigma_{s}(d,n_{p},n_{m},\lambda )$ was calculated per combination of d and $n_{p}$ with the function calcmie from MatScat^42^^,^^43^ in MATLAB (R2024a, The MathWorks Inc., Natick, Massachusetts, United States), which applies Mie theory^25^ for spherical particles in a medium with refractive index $n_{m}$ and wavelength λ.

The reconstructed scattering coefficients were calculated at λ=660  nm and $n_{m}=n_{\text{serum}}$, for all included milk samples in six sets by (1) only using the sample-specific fat concentration while keeping all other input at the arithmetic mean of all included samples, (2) to (5) using the sample-specific fat concentration and one sample-specific property (fat concentration, serum refractive index, MFG refractive index, MFG size distribution, and non-fat particle diameter and refractive index distribution) while keeping all other inputs at the arithmetic mean of all included samples, and (6) using the sample-specific information for all properties. Error margins for $\mu_{s,\text{Reconstructed}}$ in milk were set at 10%, based on the accuracy of the diverse input parameters.

### 2.4 Statistical Analysis

Finally, the similarity among the scattering coefficients from light scattering experiments and reconstructions was quantified with a linear regression analysis using the function polyfit in Matlab (R2024a, The MathWorks Inc., Natick, Massachusetts, United States) to obtain the best linear fit (least-squares). In addition, the function corr was used to calculate the Spearman correlation coefficients ρ and p-values between the experimental and reconstructed scattering coefficients.

## 3 Results

### 3.1 Sample-Specific Properties

The sample-specific properties of all 50 included milk samples are summarized in Table 1.

**Table 1:** Summary of the sample-specific properties of 50 included milk samples. Reported values are the arithmetic mean (used in calculations of the reconstructed scattering coefficient), median, IQR, minimum (min), and maximum (max).

	Arithmetic mean	Median	IQR	Min–max
Fat concentration (g/dL)	3.64	3.55	3.33	0.46 to 7.82
Refractive index serum @ 660 nm (–)	1.3452	1.3451	0.0004	1.3448 to 1.3458
Refractive index MFGs @ 660 nm (–)	1.4904	1.4901	0.0039	1.4831 to 1.5009

**Fig. 3:**
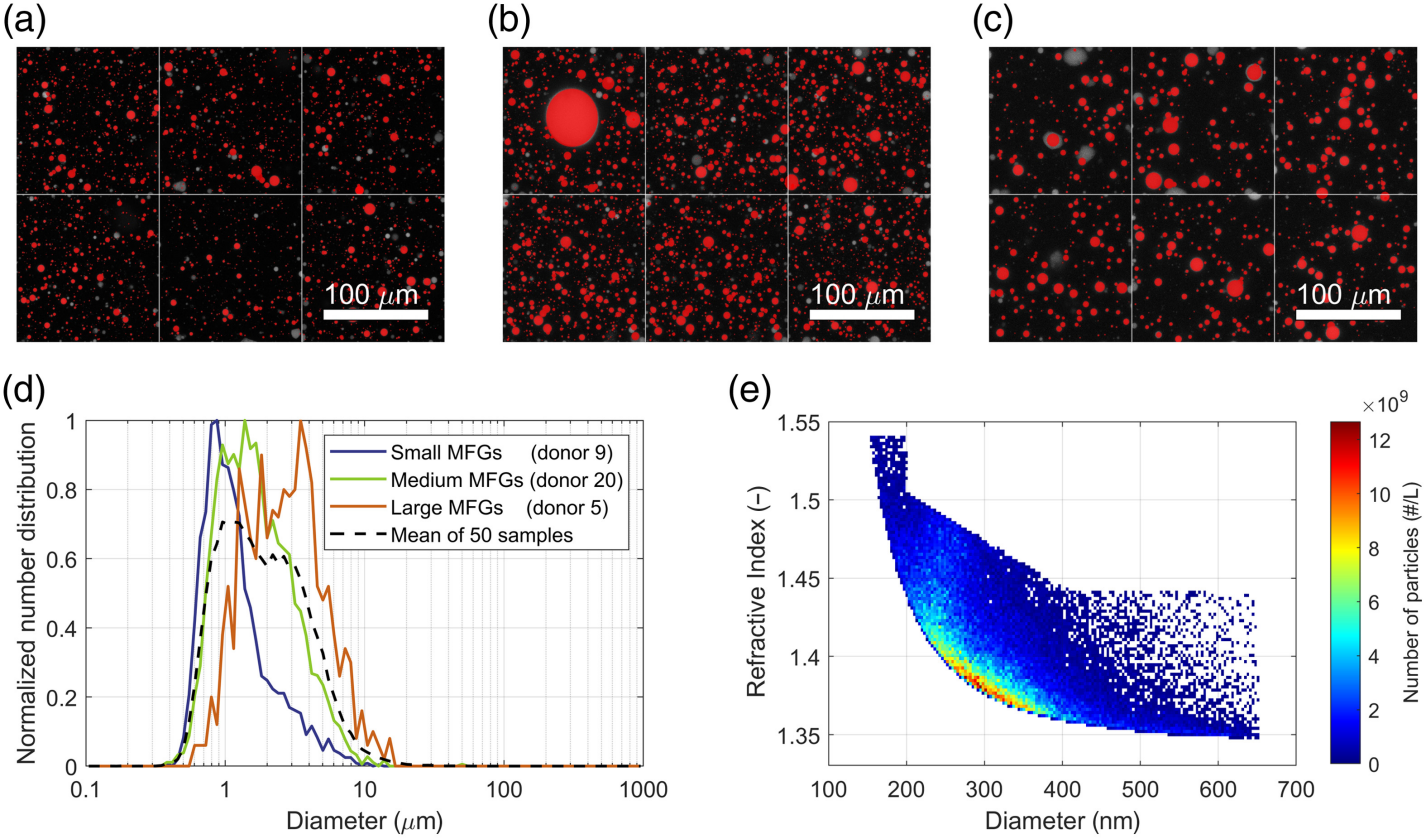
Visual representation of data used in calculations of the reconstructed scattering coefficient. (a)–(c) Maximum intensity projection of 201 stacked images from 3D confocal laser scanning microscopy with a red overlay of the detected MFGs for three representative human bulk milk samples with small (donor 9), medium (donor 20), and large (donor 10) MFGs, respectively. (d) Normalized MFG size distribution of the three representative samples and the mean size distribution of all 50 included milk samples. (e) Distribution of the diameters and refractive indices at 660 nm for the non-fat particles in a representative bulk milk sample (donor 1), measured with flow cytometry.^19^

The results from 3D confocal laser scanning microscopy with automated MFG segmentation and manual addition of large MFGs are shown in Figs. 3(a)–3(c) for three representative bulk milk samples with small, medium, and large MFGs, respectively. The resulting number distributions and mean number distribution of all included samples are given in Fig. 3(d).

The distribution of diameters and refractive indices of non-fat particles for one representative milk sample is shown in Fig. 3(e). The general shape of this distribution is representative of all included milk samples; however, the details and the total number of non-fat particles per milk volume vary per sample.

### 3.2 Experimental Scattering Coefficients

**Fig. 4:**
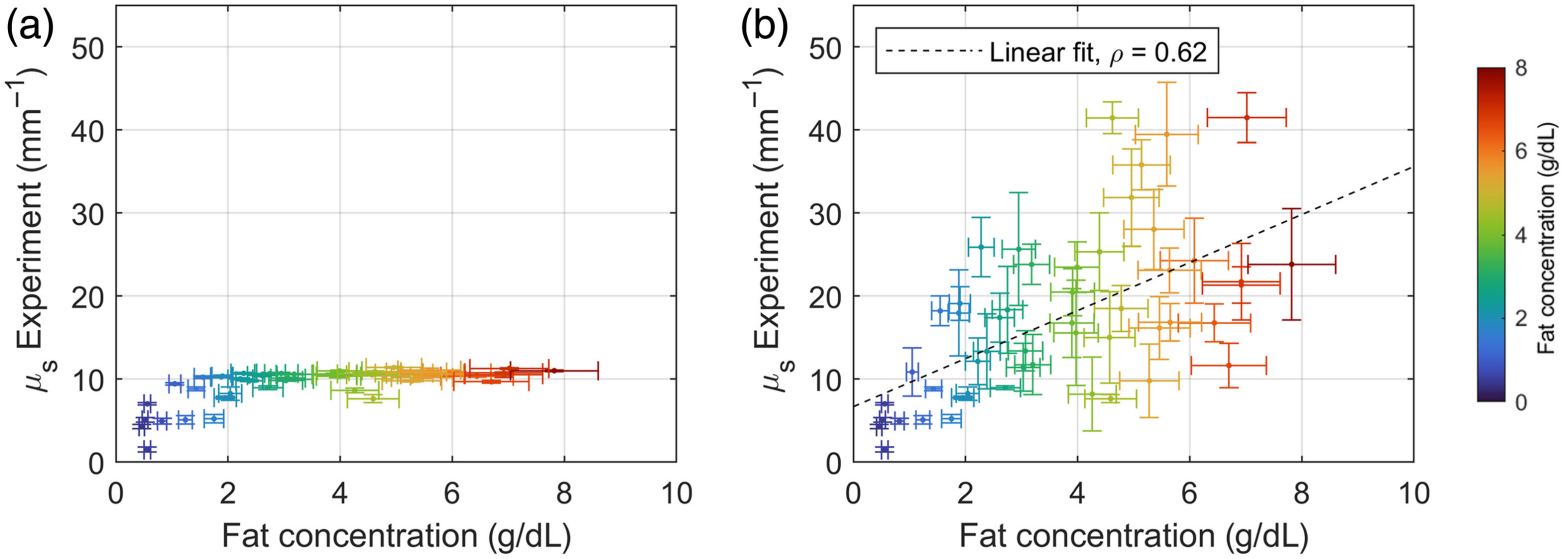
Experimental scattering coefficient for the 50 included milk samples as a function of the sample-specific fat concentration. (a) Measured in undiluted milk. Experimental scattering coefficients above $∼9.5\;{\mathrm{mm}}^{-1}$ could not be measured reliably due to multiple scattering. (b) Measured in undiluted milk (for $\mu_{s,\text{Experiment}}<8\;{\mathrm{mm}}^{-1}$) or extrapolated from measurements in diluted milk (for $\mu_{s,\text{Experiment}}\geq 8\;{\mathrm{mm}}^{-1}$). The best linear fit (least-squares) and Spearman correlation coefficient ρ are given.

The experimental scattering coefficients for the 50 included milk samples are presented as a function of the sample-specific fat concentration in Fig. 4. Twelve samples had $\mu_{s,\text{Experiment}}<8\;{\mathrm{mm}}^{-1}$, such that the scattering coefficient from undiluted milk measurements could be used. The $\mu_{s,\text{Experiment}}$ values from undiluted milk for the other 38 samples were saturated due to multiple scattering. More reliable values for $\mu_{s,\text{Experiment}}$ of these highly scattering milk samples ($\mu_{s,\text{Experiment}}\geq 8\;{\mathrm{mm}}^{-1}$) were extrapolated from measurements on diluted milk. The scattering coefficient was below $8.3\;{\mathrm{mm}}^{-1}$ for all diluted milk samples before extrapolation. The limited influence of multiple scattering in the diluted milk measurements justifies a linear extrapolation for the scattering coefficients of these samples. A moderate correlation (Spearman ρ=0.62, p<0.001) was found between $\mu_{s,\text{Experiment}}$ and the sample-specific fat concentration.

### 3.3 Reconstructed Scattering Coefficients

**Fig. 5:**
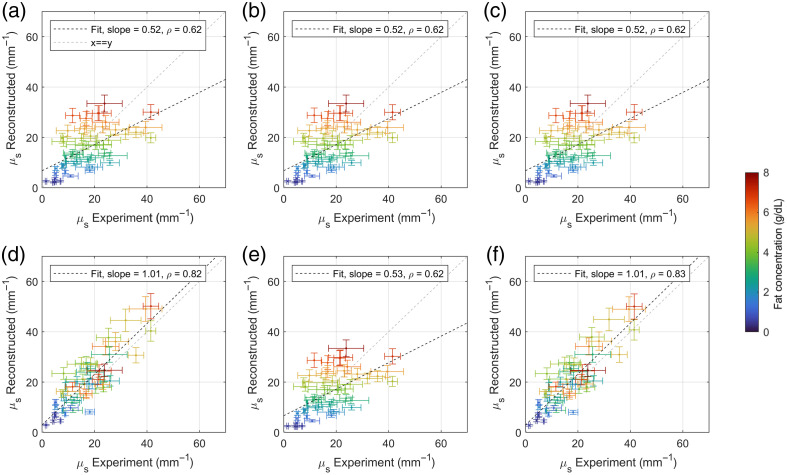
Reconstructed scattering coefficients versus experimental scattering coefficient for all 50 included milk samples. (a)–(e) Reconstructed scattering coefficient based on single sample-specific properties while keeping all other inputs at the arithmetic mean of all included samples. Varied properties are: (a) fat concentration, (b) fat concentration and serum refractive index, (c) fat concentration and MFG refractive index, (d) fat concentration and MFG size distribution, (e) fat concentration and non-fat particles. (f) Reconstructed scattering coefficient based on all sample-specific information: fat concentration, serum refractive index, MFG refractive index, MFG size distribution, and diameter and refractive information of non-fat particles. In each graph, the best linear fit (least-squares) and Spearman correlation coefficient ρ are given.

The reconstructed scattering coefficients for the 50 included milk samples are presented as a function of the experimental scattering coefficient in Fig. 5. A moderate correlation (Spearman ρ=0.62, p<0.001) can be observed between the experimental and reconstructed scattering coefficients, if only the sample-specific fat concentration is taken into account [Fig. 5(a)]. The correlation does not change noticeably when the sample-specific refractive index of the serum [Fig. 5(b)], refractive index of the MFGs [Fig. 5(c)], or non-fat particle information [Fig. 5(e)] are taken into account. Notably, the correlation improves significantly (Spearman ρ=0.82, p<0.001) when the size distribution of the MFGs is taken into account [Fig. 5(d)]. This correlation is almost equal to the correlation when all sample-specific information is taken into account (Spearman ρ=0.83, p<0.001) [Fig. 5(f)].

## 4 Discussion

In this study, we determined the influence of sample-specific milk properties on the scattering coefficient of human milk. Hereto, we compared experimental scattering coefficients with reconstructed scattering coefficients from Mie theory, based on either mean or sample-specific input. Our unique and broad dataset with various input parameters per milk sample enabled new insights into the milk properties that influence light scattering in human milk.

First, our results demonstrated that the scattering coefficient in human milk is moderately correlated with the fat concentration. This is consistent with results from other studies in human milk^13^ and bovine milk.^44^ These studies both hypothesized that the remaining variation in the optical properties of human milk is due to the biological variability in MFG size distribution.

In this study, we confirmed this hypothesis and showed that the combination of sample-specific fat concentration and sample-specific MFG size distribution indeed explained most of the variation in the scattering coefficient among human milk samples. Our results support simulations that demonstrated that the MFG size distribution influences light scattering in homogenized bovine milk.^45^^,^^46^ The significant influence of the MFG size distribution on light scattering might also explain why current optical methods for fat quantification in human or bovine milk require homogenization before analysis.^23^^,^^47^

We recognize that the MFG size distribution is a complicated variable for further analysis. It would be beneficial to reduce the MFG size distribution to simple parameters, such as the mean or median diameter, or a distribution width. However, Zhang et al.^46^ found in their simulation work that it is necessary to use the full size distribution instead of an average diameter per sample to obtain accurate scattering behavior in bovine milk. More specifically, it is important to include any large MFGs (>10  μm) in the size distributions, as they have a large influence on the scattering coefficient despite their low number concentration. The present results indicate that the manual segmentation of large MFGs in our 3D CLSM method works sufficiently for this purpose.

Next to the fat concentration and MFG size distribution, our dataset allowed us to study the sample-specific influences of the refractive index of milk serum, the refractive index of MFGs, and the refractive index and size distribution of non-fat particles. None of these three variable groups significantly influenced the reconstructed scattering coefficient when compared with the mean input of these parameters. For the refractive index of the milk serum, this could be explained by the small variation among samples [median 1.3451, interquartile range (IQR) 0.0004], which has essentially no effect on the reconstructed scattering coefficient from Mie theory. The variation in refractive index of MFGs is slightly larger (median 1.4901, IQR 0.0039), but still negligible compared with the refractive index contrast between serum and MFGs, which is used in Mie calculations. Therefore, it is sufficient to use an average value for human milk instead of the sample-specific values. Importantly, it remains essential to use the species-specific refractive index of MFGs when computing scattering coefficients. If, for example, the bovine milk fat refractive index (1.4583 at 660 nm^38^) is used instead of the mean value for human MFGs, the reconstructed scattering coefficients change up to 15%.

The sample-specific contribution of non-fat particles to the reconstructed scattering coefficient was insignificant, as these particles are small (<1  μm) and often have a low refractive index contrast compared with MFGs. The non-fat particles in high-scattering milk samples could have been destroyed by the dilution step using ultrapure water. However, the correlation coefficient between reconstructed and experimental scattering coefficients remains equal if only the 12 undiluted milk samples are included in the analysis: ρ=0.771 or ρ=0.776 respectively without or with sample-specific non-fat particle information. Our findings are consistent with those of Veenstra et al.,^48^ who found a negligible influence of casein micelles on $\mu_{s}$. However, they did find a small contribution of casein micelles to the reduced scattering coefficient $\mu_{s}^{\prime }$, which implies that sample-specific concentrations of non-fat particles in human milk could still be important in backscattering or side-scattering geometries. In our study, only mature milk (at least 10 to 15 days postpartum) was included. Future research could investigate if the scattering contribution from non-fat particles is more prominent in colostrum (milk from the first days postpartum), which generally contains more protein and less fat than mature milk.^49^ To ensure optimal preservation of non-fat particles in future investigations, we recommend using a tailored dilution medium for human milk instead of ultrapure water.^50^

Our extensive dataset on the included milk samples allowed reconstructing scattering coefficients based on measured sample-specific values with minimal assumptions about input variables. Still, a part of the variation in the experimental scattering coefficient remained unexplained. These remaining differences may be caused by measurement inaccuracies and detection limits of sample-specific properties, as well as measurement inaccuracies of the experimental scattering coefficient itself. Small differences could also have occurred between the aliquoted milk samples and sample handling for the various measurement techniques. For reconstruction, we assumed spherical MGFs with a uniform refractive index and an equal refractive index value for all MFGs in one sample. It should also be noted here that absorption was assumed to be negligible at the evaluated wavelength of 660 nm.^13^ Likewise, the influence of dependent scattering was neglected in both the reconstructions and the transmission measurements, because all milk samples, diluted when needed, had a low fat concentration (<3.0  g/dL^46^). We did not compare among fore, bulk, and hindmilk sample groups in the analysis, as any significant milk differences among these groups were already taken into account in the different properties of interest in this study.

The results of this study provide new insight into the high relevance of donor-specific MFG size distributions for studying the light scattering behavior of human milk. More generally, the sample-specific particle size distribution is likely also important for milk from other species, such as raw and/or homogenized bovine milk.

The combined influence of the fat concentration and the MFG size distribution on the scattering coefficient restricts accurate fat quantification based on forward scattering only in raw milk. The correlation between $\mu_{s}$ and the fat concentration could improve for other wavelengths, as the effect of the bovine MFG size distribution is smallest between 1300 and 1400 nm.^44^ For improved fat quantification methods in human milk, future research could compensate for the effects of the MFG size distribution by incorporating multiple wavelengths^51^ or multiple angles.^52^

## 5 Conclusion

The purpose of this study was to determine the contribution of sample-specific milk properties to the forward scattering coefficient of human milk. The combination of the sample-specific fat concentration and MFG size distribution explains most of the variation in the experimentally obtained scattering coefficients. In contrast, the sample-specific distribution of non-fat scattering particles and sample-specific refractive indices of the milk serum and MFGs have no significant influence on the reconstructed scattering coefficients. It remains important to use species-specific values of these properties to obtain accurate light scattering models for human milk. The established dataset of human milk optical properties can be expanded and utilized in future studies to support the development of optics-based measurement systems for human milk and further research on breastfeeding using non-invasive methods.

## Supplementary Material

10.1117/1.BIOS.3.1.012105.s01

## Data Availability

In compliance with the journal’s data availability policy, the values behind the reported measures and the values used to build the graphs in this article are shared as Supplementary Material.
